# Prognostic Factor Analysis of Visual Outcome after Vitrectomy for Rhegmatogenous Retinal Detachment

**DOI:** 10.3390/jcm9103251

**Published:** 2020-10-12

**Authors:** Polona Zaletel Benda, Bor Vratanar, Goran Petrovski, Ana Uršula Gavrić, Katja Matović, Ana Gornik, Katarina Vergot, Anila Lumi, Xhevat Lumi

**Affiliations:** 1 Eye Hospital, University Medical Centre Ljubljana, Grablovičeva 46, 1000 Ljubljana, Slovenia; polonazaletel@gmail.com (P.Z.B.); anaursulakosir@gmail.com (A.U.G.); katjamatovic@gmail.com (K.M.); ana.gornik@gmail.com (A.G.); katarina.vergot@gmail.com (K.V.); lumi.anila@gmail.com (A.L.); 2 Institute for Biostatistics and Medical Informatics, Faculty of Medicine, University of Ljubljana, Vrazov trg 2, 1000 Ljubljana, Slovenia; bor.vratanar@mf.uni-lj.si; 3 Center for Eye Research, Department of Ophthalmology, Oslo University Hospital and Institute of Clinical Medicine, Faculty of Medicine, University of Oslo, 0450 Oslo, Norway; goran.petrovski@medisin.uio.no

**Keywords:** rhegmatogenous retinal detachment, pars plana vitrectomy, visual outcome, discontinuity of ellipsoid zone, optical coherence tomography

## Abstract

Pars plana vitrectomy (PPV) is a surgical approach mainly chosen for complex rhegmatogenous retinal detachment (RRD) repair with highly variable functional results. The aim of this analysis was to evaluate the impact of preoperative factors and postoperative optical coherence tomography (OCT) macular findings on the functional outcome of patients undergoing primary PPV for RRD. A retrospective analysis was performed on 88 eyes of 88 patients with complex RRD managed by PPV. A swept source OCT was used to obtain images at the postoperative visit at least 6 months after PPV. Hierarchical linear regression model was used to evaluate the influence of preoperative factors related to patient, ocular clinical and postoperative OCT macular findings on functional outcomes of PPV for RRD. Duration of symptoms (*p* = 0.031) and discontinuity of the ellipsoid zone (EZ) on OCT (*p* = 0.024) showed statistically significant negative correlation, while preoperative best-corrected visual acuity (BCVA; *p* < 0.001) showed statistically significant positive correlation to postoperative BCVA. Preoperative BCVA and duration of symptoms can be used as prognostic factors for visual outcome in patients undergoing PPV for RRD. Discontinuity of the EZ was the only postoperative OCT variable related to worse postoperative visual outcome.

## 1. Introduction

Rhegmatogenous retinal detachment (RRD) is a potentially vision-threatening retinal disorder with an approximate annual incidence of 12 per 100,000 people [1]. RRD represents a medical emergency. Without appropriate treatment, permanent visual loss in the affected eye may occur [2].

Standard surgical treatment options for retinal detachment are pneumatic retinopexy, scleral buckling and pars plana vitrectomy (PPV) [3]. The appropriate surgical approach is mainly chosen according to the complexity of detachment, age of the patient and the surgeon’s preference. Pneumatic retinopexy and scleral buckling surgery are the methods of choice for simple RRD, while PPV is more widely used for complex RRD [4]. In simple RRD, the detachment is localized to a single, small retinal tear or hole at the retinal periphery accompanied by good visibility of the fundus [5]. In complex RRD, the detachment is partial, subtotal or total with multiple retinal breaks, posterior breaks, retinal dialysis or a giant tear. It can also be associated with vitreous hemorrhage, ocular trauma and proliferative vitreoretinopathy (PVR) [5,6].

With modern surgical techniques, even in complex cases a relatively high rate of anatomical success is achieved in the treatment of RRD. The primary anatomical success rate for the primary vitrectomy range from 70% to 90% [7,8] with a final anatomic success rate up to 97% [4,9]. In contrast to the anatomical outcome, functional results are highly variable. Several preoperative variables have been reported to be associated with worse functional outcome or an increased risk of surgical failure, such as the duration of symptoms [10,11], preoperative best-corrected visual acuity (BCVA) [10,12,13], extent of retinal detachment, number of retinal quadrants involved [14], previous lens extraction or intraocular surgery other than RRD, high myopia, preoperative hypotony [8] and the presence of PVR [10,14,15]. To date, numerous reports have attempted to link the functional outcome of surgical treatment to preoperative variables [10,11,12,13,14,15]. Modern retinal imaging techniques provides a detailed insight into the morphological condition of the retina in the area of the macula lutea, the condition of which has a large impact on the functional outcome of surgical treatment [16,17,18,19,20,21,22,23,24,25,26,27,28,29]. The structural condition of the macula can be influenced by preoperative and intraoperative and postoperative factors.

The aim of our retrospective analysis was to evaluate the impact of preoperative factors and postoperative optical coherence tomography (OCT) macular findings on the functional outcome of patients undergoing primary PPV for complex RRD.

## 2. Methods

### 2.1. Patient Selection

This study used retrospective data collection of patients treated primarily by small gauge PPV for complex retinal detachment between January and December, 2016 at the Eye Hospital, University Medical Centre Ljubljana, Ljubljana, Slovenia. The study design and data analysis were in accordance with the tenets of the Declaration of Helsinki of 1989. Written informed consent from all patients was obtained before surgical procedure.

Eighty-eight (88) patients (88 eyes) who underwent PPV for RRD were included in this study. The inclusion criterion was: patients treated primarily by vitrectomy for complex RRD. Patients with RRD managed by scleral buckle, pneumatic retinopexy or combined PPV and scleral buckle were excluded. In addition, retinal detachments related to previous eyeball trauma and patients with other retinal pathology potentially affecting macular function were also excluded. Patients who had retinal cryopexy during PPV were also excluded. All patients had at least one follow-up appointment at the Eye Hospital and they completed both visual acuity assessment and OCT imaging of the macula. All surgeries were performed by one experienced retinal surgeon (XL).

### 2.2. Data Collection

Preoperative data collected from the patient’s records were: age at the time of surgery, gender, axial length (AL) of the operated eye, duration of symptoms, BCVA, presence of crystalline lens or intraocular lens (IOL), macular status (on/off) and presence of PVR grade C1 or higher. PVR stage was graded according to the updated classification of Retina Society Terminology Committee (1991) [30].

In retinal detachments with clear optic media and macula on, the AL measurement was provided by the IOL Master Optical Biometer (Carl Zeiss Meditec AG, Jena, Germany). In detachments with opaque optical media or macula off, A-scan ultrasound biometry was performed using 10 MHz frequency probe. Intraoperative variable that was recorded included the choice of tamponade (air, type of gas or silicon oil).

A Topcon swept source optical coherence tomography (SS-OCT) was used to obtain all OCT images (Swept source OCT, DRI OCT Triton, Topcon, Tokyo, Japan). The horizontal cross-sectional B scan image was recorded at the postoperative visit at least 6 months after the surgery. Central retinal thickness (CRT) was measured using the software-based measurement tool. Recorded were also the presence of the epiretinal membrane (ERM), macular hole, discontinuity of the ellipsoid zone (EZ) and cystoid macular edema (CME). Patients were diagnosed with CME if the central macular thickness (central 1000 μm wide region) was ≥300 μm on SS-OCT imaging [31] or intraretinal (intraretinal edema) or subretinal (serous retinal detachment) fluid was detected on OCT [32]. ERM was recorded as well as any presence of irregular and hyper-reflective layer over the internal limiting membrane, regardless of whether there were signs of retinal wrinkling or not. These data were evaluated based on the consensus of OCT definition of ERM by Hubschman et al. [33]. At the same postoperative visit, functional outcomes were recorded by determining BCVA. The need for additional secondary surgical intervention was also recorded.

### 2.3. Surgical Procedure

The surgical procedure was performed under either regional or general anesthesia. A 3-port PPV was performed in all patients, using 23 or 25-gauge instrumentation and non-contact wide-angle viewing system. Trocars were placed in a way that allows peripheral vitrectomy to be performed without touching the crystalline lens, and also switching between the 3 entry sites, if necessary. The arrangement of sclerotomy sites in combination with 29-gauge chandelier endoillumination and bimanual work, allowed for safe shaving of the peripheral vitreous. Endolaser photocoagulation using curved probe was applied around the retinal tear or 360° to retinal periphery. At the end of the surgery, the air–gas mixture (either 20% sulfur hexafluoride-SF_6_ or 10–15% perfluoropropane-C_3_F_8_) or silicon oil (SO, 2000 Centistokes) endotamponade were used. SO was used in patients with RRD in the only functional eye, in patients with extended retinectomies or giant tears, and in those who were unable to maintain a facedown posture after surgical procedure. When the air or gas mixture was used for endotamponade, patients were asked to maintain a facedown posture for a week. The target period for the removal of SO tamponade was set at approximately 3 months. Combined cataract surgery and vitrectomy was performed if visualization of the retina was not sufficient because of lens opacity. PPV as a single surgical approach was performed in 80 patients and in 8 patients a combined surgical approach was performed (PPV, phacoemulsification and IOL implantation in capsular bag). In 5 patients, cataract surgery was performed during follow-up or at the occasion of SO removal. SO was removed approximately 3 months after primary surgery.

Surgery was considered successful only in cases when the retina remained completely attached at six months follow-up after a single procedure in eyes treated with PPV and gas tamponade or six months after SO removal in eyes with PPV and SO tamponade.

### 2.4. Statistical Analysis

The frequencies for categorical variables, and the means and standard deviations for numerical variables were calculated. To estimate the effect of preoperative and postoperative factors on postoperative BCVA, the data were analyzed using hierarchical linear regression. In the first step, preoperative patient-related factors were included. In the second step, the preoperative factors related to the patient and ocular clinical findings were added to the previous model; in the third step, postoperative factors were included. At each step, the fit of the model was examined graphically. To improve model fit, preoperative and postoperative BCVA were transformed to decimal notation (Snellen decimal units, see Supplementary Materials Figure S1 for model fit). All three models fitted the data adequately. F-test was used to test the statistical significance of the model, and *t*-tests were used to test the statistical significance of estimated parameters. Nested models were compared using the *F*-test. At each step, the adjusted proportion of variance explained (R^2^) was also reported. Lastly, in the Supplementary Materials we also presented the relationship between covariates and predicted BCVA values measured in logMAR units (see Supplementary Materials Figures S2 and S3). Statistical analysis was performed with R version 3.5.1. (R Core Team 2018, Vienna, Austria) [34]. The significance level was set at α = 0.05 for all statistical tests. There were no missing values in the data set used.

## 3. Results

### 3.1. Baseline Characteristics

A total of 88 eyes of 88 patients characterized by male predominance (60 males, 28 females; sex ratio: 2.1) who underwent PPV for RRD were enrolled in this study. The mean age of all patients at the time of the surgical procedure was 59.9 y ± 1.4 SD (range: 18–85 y). The mean age of the male patients was 59.8 y ± 12.5 SD (range 18–84 y), and of the female patients 60.4 y ± 12.4 SD (range: 25–85 y; Table 1) Patients’ characteristics are shown in Table 1.

Descriptive statistics for preoperative and postoperative factors (OCT macular findings) studied is shown in Table 2 for descriptive variables, and in Table 3 for numerical variables.

**Figure 1:**
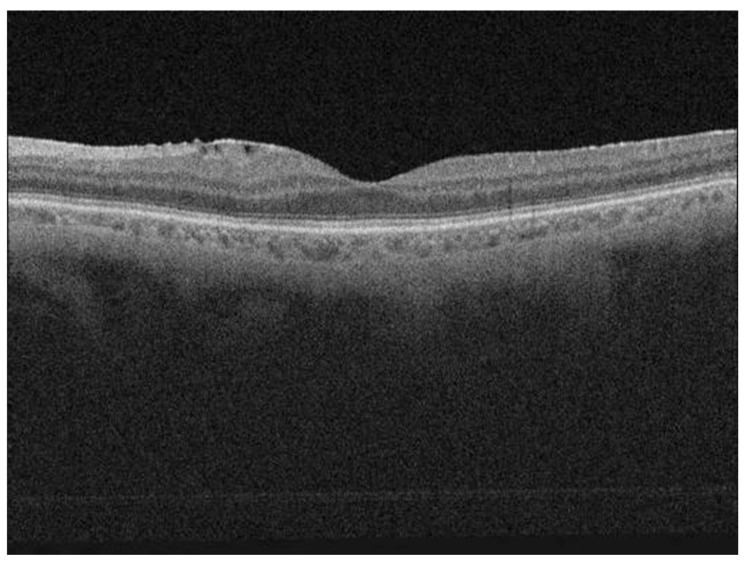
Postoperative optical coherence tomography (OCT) scan of a patient showing a well restored ellipsoid zone (EZ); the scan is showing also the epiretinal membrane.

The cohort of patients studied beside male predominance showed the presence of a higher phakic rate. More than two thirds of patients presented with macula-off RRD (68 patients; 77%), and one third with the presence of at least grade C1 PVR (29 patients; 33%). The reported duration of symptoms was highly variable, with the average of 28 days ± 70.2 SD (range: 0–550 days). Preoperative BCVA was 1.71 logMAR (±1.24 SD) and postoperative BCVA 0.58 logMAR (±0.71 SD). The average follow-up period was 11.7 months (range 6–24 months). The primary anatomical success rate after vitrectomy was 93% (82/88 patients); six patients needed additional surgery due to redetachment. After the second surgery, final retinal reattachment was achieved in all 88 eyes. The majority of eyes received gas or air endotamponade: 25 patients (28%) received 15% C_3_F_8_; 30 patients (34%) 10% C_3_F_8_; 13 patients (15%) 20% SF_6_ and 2 patients (2%) air. In the primary surgery, 18 patients (21%) received SO endotamponade and 4 patients with gas endotamponade and redetachment after primary surgery received SO as endotamponade in the second intervention. The SO was removed from the eye in all patients approximately 3 months after the surgery. Postoperative OCT macular analysis showed CME in 13 patients (15%), macular hole in 2 patients (2%) and discontinuity of EZ in 34 patients (39%) ERM was noted in 47 patients (53%), however, when these data were evaluated according to the ERM definition by Hubschman et al., the ERM was present in 2 patients (2%) [31] (Figure 1).

### 3.2. The Effect of Preoperative Factors on Postoperative BCVA

Hierarchical linear regression was used to predict the postoperative BCVA based on different preoperative factors. In the first step, preoperative patient-related factors were included. The influence of the patients’ age, duration of symptoms and preoperative BCVA are shown in Table 4. Duration of symptoms (*p* = 0.031, *t*-test) and preoperative BCVA (*p* < 0.001, *t*-test) showed a statistically significant correlation to postoperative BCVA, with negative correlation between duration of symptoms and postoperative BCVA, and positive correlation between preoperative and postoperative BCVA.

In the second step, preoperative factors related to patient and ocular clinical findings were included as predictor variables. The influence of the patients’ age, duration of symptoms, preoperative BCVA, axial length, lens status, macular status (on/off) and the presence of PVR are shown in Table 5. Preoperative BCVA was the only preoperative factor that showed statistical significance related to functional outcome in the second model (*p* = 0.001, *t*-test).

The first (F_3,84_ = 7.11, *p* < 0.001, R^2^ = 0.17) and the second model (F_7,80_ = 3.58, *p* = 0.002, R^2^ = 0.17) were statistically significant. However, the difference between the two models was not statistically significant (F_4,80_ = 0.94, *p* = 0.444). Therefore, no evidence could be found that the preoperative factors related to ocular clinical findings explain any additional variance above predictors already included in the first model.

### 3.3. The Effect of Preoperative Factors and Postoperative OCT Macular Findings on Postoperative BCVA

**Figure 2:**
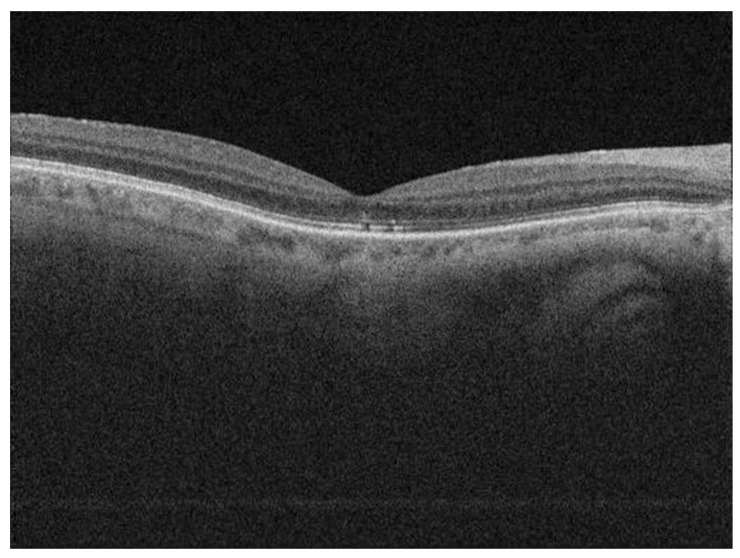
Postoperative OCT scan of a patient showing discontinuity of EZ.

When preoperative factors (patients’ age, axial length, lens status, duration of symptoms, preoperative BCVA, macular status and PVR) and postoperative OCT macular findings (CRT, presence of ERM or CME and status of the EZ (Figure 1 and Figure 2)) were included in the model, the preoperative BCVA (*p* = 0.011, *t*-test) appeared to be a statistically significant prognostic factor, while the status of the EZ (*p* = 0.024, *t*-test) showed statistically significant correlation with postoperative BCVA. Patients with discontinuity of the EZ on OCT after vitrectomy (Figure 2) had statistically significant worse postoperative BCVA (Table 6). The third model is also statistically significant (F_11,76_ = 3.44, *p* < 0.001, R^2^ = 0.24). In the third step, the proportion of variance was statistically significantly higher than in the previous step (F_4,76_ = 2.67, *p* = 0.038).

## 4. Discussion

A better modern understanding of the pathological mechanisms in the process of retinal detachment formation, better surgical approaches and technical advances that allow for more sophisticated, accurate and less traumatic surgical treatment have greatly improved the anatomical success rate in RRD management. Despite a high anatomic success rate in patients undergoing PPV for primary RRD, the functional outcome is highly variable. Mostly, as prognostic factors for a visual outcome in previous studies, preoperative variables have been analyzed. Previous reports have found the postoperative visual acuity outcome to be related to the preoperative status of the macula [8,10], preoperative BCVA [10,12,13], the duration of symptoms [10,11], the extent of retinal detachment, number of retinal quadrants involved [14], previous lens extraction or intraocular surgery other than RD, high myopia, preoperative hypotony [8] and presence of PVR [10,14,15].

Advances in retinal imaging using OCT have shown possible causes for worse functional outcome in RRD surgery despite a successful anatomical outcome. Reduced postoperative BCVA may result from ERM, CME, macular hole, the presence of subretinal fluid and distortion or discontinuity of EZ [14]. Several studies have reported the use of spectral domain OCT in the assessment of follow-up in patients undergoing surgical repair for RRD [16,17,18,19,20,21,22,23,24,25,26,27,28,29].

In this retrospective study, 88 eyes undergoing PPV for primary RRD were analyzed. Our results suggest that the duration of symptoms, preoperative BCVA and the integrity of the EZ detected on OCT imaging are statistically significantly correlated to postoperative BCVA. Hierarchical regression analysis emphasized that among these prognostic factors, the preoperative BCVA had the strongest statistical significance.

The duration of symptoms was significantly associated with functional outcome also in the study of Pastor et al., where 517 eyes undergoing PPV or scleral buckling for RRD were analyzed. Their results showed that a shorter duration of symptoms correlated to better final BCVA. In patients with BCVA > 20/40, the mean duration of symptoms was 10.8 days, while in patients with a final VA between 20/50 and 20/100 it was 13.9 days, and in those with a final VA lower than 20/100 it was 27.9 days [10]. The latter is also similar to our study’s results (27.9 days). A similar effect of symptoms duration on the functional outcome after PPV for macula-off RRD was reported by Kim et al. in 81 patients. The duration of symptoms of 6 days or less was related to better postoperative BCVA than in patients with longer duration of symptoms [11].

Our hierarchical regression model showed that eyes with better preoperative BCVA achieved better functional outcome after PPV. Although preoperative BCVA was not included as prognostic factor in the Pastor and Gerding studies, their results suggest that the functional outcome is in relation to the preoperative status of the macula, and the latter is closely associated to the preoperative BCVA [10,12]. Suzuki et al. presented 56 eyes with the macula off RRD undergoing PPV or scleral buckling and demonstrated that postoperative BCVA 6 months after the surgery was in positive correlation to preoperative BCVA [13].

Postoperative OCT macular findings in our study reported a macular hole in 2%, CME in 15% and discontinuity of the EZ zone in 39% of the patients. In previous studies, distortion of EZ was found in 40% in Cho’s study performed on 12 patients [17], and in 53% in Delolme’s study performed on 30 patients [23]. Schocket et al. reported higher percentages of EZ distortion (82%) performed on 17 patients [16]. However, these studies were conducted on a much lower number of patients compared to our study [16,17,22]. Since we primary registered every hyper-reflective layer on the inner surface of the retina as ERM, we recorded ERM changes in 53% of patients. This variable did not show any statistically significant impact on the postoperative visual outcome. After evaluation according to the definition of ERM made by Haubschman et al., however, we found true ERM with traction on macula in only 2% of patients [33].

Our analysis from postoperative OCT findings only found that the integrity of the EZ showed statistically significant correlation with the postoperative BCVA. In line with our findings, the PIONEER study conducted on 15 patients showed that postoperative quantitative integrity of EZ at 12 months is directly correlated with visual outcomes [29]. Similar results were obtained by Cheng et al. in 43 patients with 6 months minimal follow-up time. Besides that, the macula off group was associated with higher postoperative visual gains than the macula on group [35]. Kobayashi et al. retrospectively analyzed 29 eyes after successfully reattached RDD undergoing PPV. Their study showed that BCVA and the integrity of the EZ 2 weeks after vitrectomy were independent prognostic factors of the final BCVA 12 months after the surgery [26]. Dell’Omo and Terauchi et al. reported significant correlation between the thickness of the EZ-RPE complex and BCVA 1 month postoperatively [27,28]. In addition to the integrity of EZ, Gharbiya et al. emphasized that the integrity of the intermediate line and the outer nuclear layer thickness changes on OCT imaging may be important predictors of functional outcome after anatomically successful RRD surgery with the scleral buckle [20].

Preoperative factors that were analyzed in our study but showed no significant associations with functional outcome included patients’ age, axial length, lens status, macular status (on/off) and PVR status. In contrast to our results, several previous studies have confirmed correlation of these preoperative factors with the functional outcome after anatomically successful surgery. Pastor et al. showed that age of the patients correlated inversely with the final functional outcome; patients with final VA > 20/100 were younger (53.2 y) compared to patients with final VA lower than 20/100 (59.6 y) [10]. The mean age in our cohort of patients (59.9 years) was similar to the group of patients with lower final VA reported in the study of Pastor et al. [10].

Our analysis of the lens status (phakic, pseudophakic or aphakic) showed no correlation to the visual outcome, and the majority of patients were phakic (60%). The lens status also showed no impact on the functional outcome in the study of Pastor et al. However, vitrectomy in their study was mostly performed in pseudophakic eyes and resulted in a worse final visual acuity [10].

In contrast to our results, where no statistical correlation between the macular status (on/off) and postoperative BCVA could be shown, Gerding et al. and Pastor et al. [10,12] reported worse postoperative visual outcome in macula-off RRD. The number of macula-off RRD in our cohort of patients at the time of surgery was similar to both these studies (77% in ours versus 80% in Gerding’s, and 65% in Pastor’s study) [10,12]. Lecleire-Collet et al. have found the height of detached retina at the central fovea and the distance from the central fovea to the nearest undetached retina in macula-off detachments to be negatively correlated to the final postoperative visual acuity [36]. Similar findings were reported by Joe SG et al. [37]: they found the final BCVA to be correlated with retinal detachment height and the integrity of the junction between the photoreceptor inner and outer segments combined with the continuity of the external limiting membrane. On the other hand, in the same study on 31 patients with the mean duration of macula-off detachment of 15.5 ± 15.2 days, the visual acuity outcome showed no correlation with the RRD duration [37]. In contrast, the results of multiple regression analysis in the study of Suzuki et al. on 56 patients with the average duration of macula-off detachment of 33.3 ± 72.7 days revealed that the duration of macular detachment and total cross-sectional macular area were the only independent factors predicting the 6-months postoperative BCVA [13]. It is expected that the greater distance of foveal cones from the retinal pigment epithelium and choriocapillaris, the less oxygenation and nutrition they receive, and the apoptosis in these cells increases accordingly [38]. These processes are, however, far more complex and do not follow the course of simple mechanistic patterns. The height of the detached macula is indeed not stable, it changes during the day and the simple measurement at a time does not show the full picture of the position of the macula during a 24-h time. In longer duration retinal detachment cases, the situation becomes even more complex. Furthermore, retinal detachment involves besides inflammatory cells, several cytokines, chemokines and growth factors expressing either cytotoxic or protective effect upon photoreceptors [38].

Differences in the outcomes of predictive models for postoperative visual acuity between studies using different inclusion criteria are therefore expected. We believe the results of our study are useful in patients with complex RRD and longer duration of retinal detachment.

While PVR was not recognized as a prognostic factor in our hierarchical model, PVR grade C has been identified as such a factor predicting a worse visual outcome in Pastor’s and Wickham and Mitry’s statistical model [10,14,15]. Mitry et al. reported more than 2-fold increased risk of surgical failure when preoperative PVR was present [14]. The proportion of eyes graded with PVR in these studies was similar to ours (33% in our study versus 24–43% in Pastor’s, 29% in Wickham’s and 36.5% in Mitry’s study) [10,14,15].

There are some limitations in our study. Many predictor factors have been included in the regression model for a relatively small cohort of patients, most of them being statistically insignificant. Furthermore, the study was retrospective, therefore, OCT macular imaging was performed only during regular postoperative follow-up (on average 11.7 months postoperatively). However, in contrast to other reports with groups of patients managed by different surgical techniques, all patients of our group were managed by small gauge PPV. We excluded patients managed by scleral buckle, pneumatic retinopexy, cryopexy and those with combined PPV and scleral buckle. By having only patients managed with similar intraoperative approach, we believe it reduces the possibility of impact of different surgical methods on the results. Further studies on a larger number of patients with more detailed retinal imaging evaluation are needed for better definition of prognostic factors for patients undergoing PPV for RRD.

## 5. Conclusions

The results of our retrospective study indicate that preoperative BCVA and duration of symptoms to be statistically significant prognostic factors for the final visual outcome of patients undergoing vitrectomy for complex RRD. Postoperative discontinuity of EZ on OCT macular imaging was the only postoperative OCT variable related to a worse postoperative visual outcome. Although reported as preoperative prognostic factors in previous studies, age of the patients, axial length of the eye, lens status, macular status and PVR status showed no statistically significant association with the functional outcome.

## Figures and Tables

**Table 1:** Patients’ characteristics.

Gender (*n*)	Age (y)	Macular Status	Preop. BCVA (logMAR)	Postop. BCVA (logMAR)
Female (28)	60.4 (range: 25–85 y)	On: 8 (29%) Off: 20 (71%)	2.05 ± 1.29 SD	0.67 ± 0.83 SD
Male (60)	59.8 (range: 18–84 y)	On: 12 (20%) Off: 48 (80%)	1.55 ± 1.20 SD	0.54 ± 0.65 SD

**Table 2:** Pre- and post-operative ocular variables of patients included in the study.

Factors Studied	Frequency (%)
**Preoperative factors**	
*Lens status*	
Phakic	53 (60.2%)
Pseudophakic	32 (36.4%)
Aphakic	3 (3.4%)
*Macular status*	
On	20 (23%)
Off	68 (77%)
*PVR status*	
No PVR	59 (67%)
PVR (any grade C)	29 (33%)
**Postoperative factors**	
*OCT—presence of CME*	
No	75 (85%)
Yes	13 (15%)
*OCT—presence of ERM*	
No	39 (44.3%)
Yes	47 (53.4%)
Macular hole	2 (2.3%)
*OCT—discontinuity of ellipsoid zone*	
No discontinuity	54 (61%)
Discontinuity	34 (39%)

**Table 3:** Numerical variables of patients included in the study.

Factors Studied	Average	SD	Range
**Preoperative factors**			
Duration of symptoms (days)	27.9	70.2	0–550
Axial length (mm)	24.7	2.0	21.2–32.4
Preoperative BCVA (logMAR)	1.7	1.2	0–4
**Postoperative factors**			
Postoperative BCVA (logMAR)	0.6	0.7	0–3
OCT central retinal thickness (µm)	274.0	75.5	113–606

**Table 4:** The effect of preoperative patient-related factors on postoperative best-corrected visual acuity (BCVA).

	*b*	*SE*	*p*	95% CI
Patients’ age	−0.001	0.002	0.828	−0.006 to 0.004
Duration of symptoms	−0.001	0.0004	0.031 *	−0.002 to 0.000
Preoperative BCVA	0.396	0.098	<0.001 *	0.202 to 0.591

Note: BCVA was measured in Snellen decimal units. 95% CI—95% confidence interval; * *p* < 0.05.

**Table 5:** The effect of preoperative factors related to patient and ocular clinical findings on postoperative BCVA.

	*b*	*SE*	*p*	95% CI
Patients’ age	−0.002	0.003	0.538	−0.007 to 0.004
Duration of symptoms	−0.001	0.0005	0.057	−0.002 to 0.000
Preoperative BCVA	0.466	0.140	0.001 *	0.188 to 0.743
Axial length	0.000	0.017	0.979	−0.033 to 0.033
Lens status	0.094	0.067	0.167	−0.040 to 0.227
Macular status	0.125	0.105	0.236	−0.084 to 0.335
Presence of PVR	−0.031	0.073	0.667	−0.176 to 0.113

Note: BCVA was measured in Snellen decimal units. 95% CI—95% confidence interval; * *p* < 0.05.

**Table 6:** The effect of preoperative factors and postoperative OCT macular findings on postoperative BCVA.

	*b*	*SE*	*p*	95% CI
Patients’ age	−0.002	0.003	0.364	−0.008 to 0.003
Duration of symptoms	−0.001	0.0005	0.205	−0.002 to 0.000
Preoperative BCVA	0.369	0.141	0.011 *	0.089 to 0.650
Axial length	−0.004	0.016	0.791	−0.037 to 0.028
Lens status	0.117	0.068	0.091	−0.019 to 0.254
Macular status	0.053	0.104	0.612	−0.154 to 0.259
Presence of PVR	0.031	0.072	0.672	−0.113 to 0.175
OCT—CRT	0.000	0.0005	0.677	−0.001 to 0.001
OCT—presence of ERM	−0.065	0.064	0.312	−0.193 to 0.062
OCT—status of EZ	−0.174	0.076	0.024 *	−0.325 to −0.024
OCT—presence of CME	−0.103	0.102	0.318	−0.306 to 0.101

Note: BCVA was measured in Snellen decimal units. 95% CI—95% confidence interval; * *p* < 0.05.

## Supplementary Materials

The following are available online at https://www.mdpi.com/2077-0383/9/10/3251/s1, Figure S1: (A) Fit of the final model (BCVA measured in logMAR units). (B) Fit of the final model (BCVA measured in Snellen units); Figure S2: Predicted BCVA values measured in logMAR units for a level 0 and level 1 on each dichotomous covariate where other covariates in the model are fixed to the median (in case of continuous covariates) or mode (in case of dichotomous covariates); Figure S3: Predicted BCVA values measured in logMAR units for a range of values on each continuous covariate where other covariates in the model are fixed to the median (in case of continuous covariates) or mode (in case of dichotomous covariates).
