# Supplementary material for: Prognostic Factor Analysis of Visual Outcome after Vitrectomy for Rhegmatogenous Retinal Detachment
Source: J Clin Med. 2020 Oct 12;9(10):3251. doi: 10.3390/jcm9103251 (PMC7601405; doi:10.3390/jcm9103251)
Supplement: Supplementary file 1 [file jcm-09-03251-s001.pdf]

SUPPLEMENTARY MATERIALS

Model Fit

The fit of the final model was inadequate when BCVA was measured in logMAR units. The fit of the model was improved by transforming logMAR units to Snellen units (see Supplementary Figure 1 below).

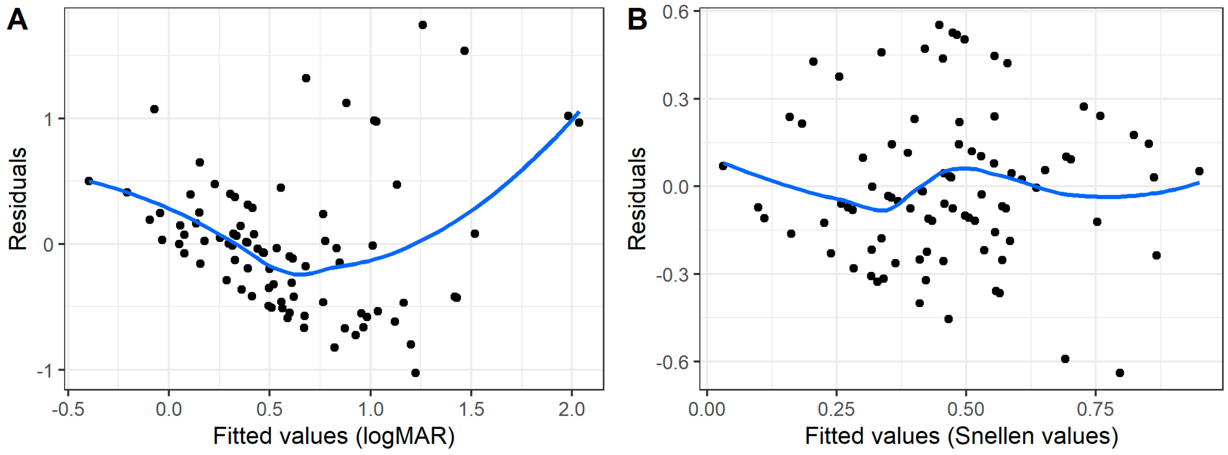

Supplementary Figure S1. A) Fit of the final model (BCVA measured in logMAR units). B) Fit of the final model (BCVA measured in Snellen units).

Predicted values (logMAR units)

The relationship between covariates and predicted BCVA values *measured in logMAR units* might also be of interest. We considered the final model only. As BCVA values measured in logMAR units are calculated via non-linear transformation of BCVA values measured in Snellen units, one cannot assess the size of the effect of the covariates on BCVA (measured in logMAR units) directly from0 Table 5 in the manuscript. Therefore, a graphical presentation of the relationship between the transformed predicted values on BCVA and each covariate is shown, where other covariates in the model are fixed to median (in case of continuous covariates) or mode (in case of dichotomous covariates). The relationship between covariates and predicted BCVA values measured in logMAR units is shown in Supplementary Figure 2 below. The relationship between continuous covariates and predicted BCVA values measured in logMAR units is shown in the Supplementary Figure S3.

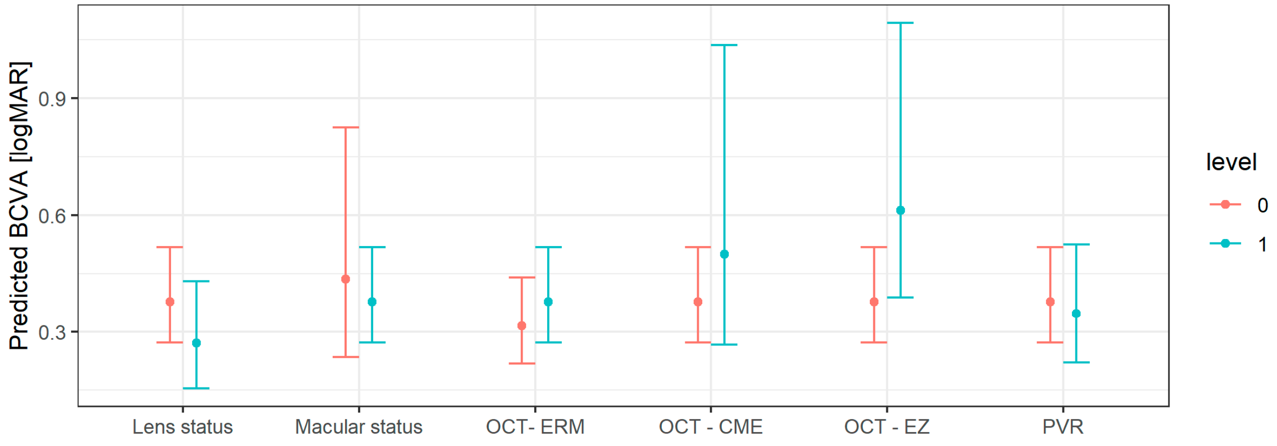

Supplementary Figure S2. Predicted BCVA values measured in logMAR units for a level 0 and level 1 on each dichotomous covariate where other covariates in the model are fixed to median (in case of continuous covariates) or mode (in case of dichotomous covariates). Lens status: 0 = phakic, 1 = pseudophakic; macular status: 0 = on, 1 = off; OCT-ERM: 0 = absent, 1 = present; OCT-CME: 0 = absent, 1 = present; OCT – EZ: 0 = No discontinuity, 1 = Discontinuity; PVR: 0 = no PVR, 1 = PVR (any grade C).

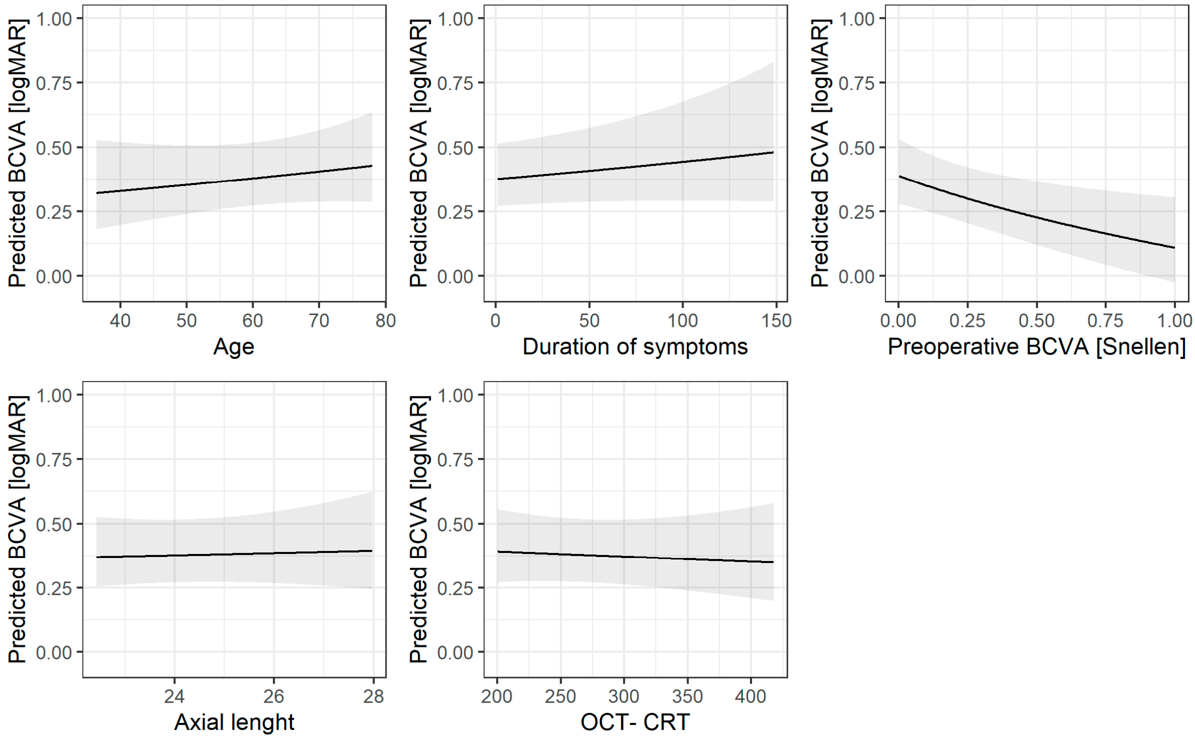

Supplementary Figure S3. Predicted BCVA values measured in logMAR units for a range of values on each continuous covariate where other covariates in the model are fixed to median (in case of continuous covariates) or mode (in case of dichotomous covariates).
